# Stability of Naked Nucleic Acids under Physical Treatment and Powder Formation: Suitability for Development as Dry Powder Formulations for Inhalation

**DOI:** 10.3390/pharmaceutics15122786

**Published:** 2023-12-16

**Authors:** Tomoyuki Okuda, Maki Okazaki, Akihiko Hayano, Hirokazu Okamoto

**Affiliations:** Faculty of Pharmacy, Meijo University, 150 Yagotoyama, Tempaku-ku, Nagoya 468-8503, Japan; g0773313@ccalumni.meijo-u.ac.jp (M.O.); g0673144@ccalumni.meijo-u.ac.jp (A.H.); okamotoh@meijo-u.ac.jp (H.O.)

**Keywords:** nucleic-acid-based medicines, physical stress, powder formation, stability, dry powder inhaler (DPI) formulations, plasmid DNA (pDNA), small interfering RNA (siRNA), naked nucleic acid formulations, spray drying (SD), spray freeze drying (SFD)

## Abstract

A number of functional nucleic acids, including plasmid DNA (pDNA) and small interfering RNA (siRNA), have been attracting increasing attention as new therapeutic modalities worldwide. Dry pDNA and siRNA powder formulations for inhalation are considered practical in clinical applications for respiratory diseases. However, physical stresses in the powder-forming process may destabilize nucleic acids, particularly when vectors with stabilizing effects are not used. We herein compare the stability of naked pDNA and siRNA through various physical treatments and two powder-forming processes. The structural and functional integrities of pDNA were markedly reduced via sonication, heating, and atomization, whereas those of siRNA were preserved throughout all of the physical treatments investigated. Spray-dried and spray-freeze-dried powders of siRNA maintained their structural and functional integrities, whereas those of pDNA did not. These results demonstrate that siRNA is more suitable for powder formation in the naked state than pDNA due to its higher stability under physical treatments. Furthermore, a spray-freeze-dried powder with a high content of naked siRNA (12% of the powder) was successfully produced that preserved its structural and functional integrities, achieving high aerosol performance with a fine particle fraction of approximately 40%.

## 1. Introduction

A number of functional nucleic acids, such as plasmid DNA (pDNA), messenger RNA (mRNA), antisense oligonucleotide, and small interfering RNA (siRNA), have been actively examined to assess their utility for innovative therapies against lethal and intractable gene-related diseases. Some have been approved for clinical use in this decade, and vaccines against coronavirus diseases 2019 (COVID-19), mainly containing mRNA as the active ingredient, were administered to more than five billion people worldwide by November 2023 [1]. Therefore, further advances in nucleic-acid-based drugs are highly anticipated; however, their safety is still a matter of concern. Viral or non-viral vectors have been adopted for many nucleic-acid-based drugs to achieve their effective transfection into the body because foreign nucleic acids are rapidly degraded by nucleases and are also challenging to transfer into target cells due to their macromolecular and highly hydrophilic properties. Lipid nanoparticles (LNPs) are used as non-viral vectors in approved mRNA and siRNA drugs, and they are considered to be safer than viral vectors [2]. However, major adverse reactions (e.g., anaphylaxis and Bell’s palsy), as well as common side effects (e.g., fever, fatigue, headache, and swelling at the injection site), are associated with mRNA vaccines, which may be related to LNPs [3,4]. As alternative strategies to these vector applications, the chemical modification and local application of naked nucleic acids are expected for increased safety, some of which (e.g., Givlaari^®^ as siRNA and Collategene^®^ as pDNA) have already been approved for clinical use [5]. Givlaari^®^ is effectively delivered to liver parenchymal cells after subcutaneous injection via N-acetylgalactosamine conjugation, escaping nuclease degradation in the blood by 2′-O-methyl, 2′-deoxy-2′-fluoro, and phosphorothioate modification. On the other hand, Collategene^®^ has no chemical modification and is directly injected into muscle tissue, a target site. Such delivery strategies are essential for naked nucleic acids to overcome their pharmacokinetic and pharmacodynamic issues, such as low biostability, targetability, and transfection efficiency.

The development of nucleic-acid-based drugs as inhaled formulations is considered promising in clinical applications for respiratory diseases for a number of reasons, such as their direct and non-invasive delivery into the lungs and the low activity of nucleases in the lungs [6]. Previous studies demonstrated that the pulmonary delivery of naked nucleic acids through intratracheal or intranasal administration to small animals successfully achieved gene transfection and therapeutic effects in the lungs [7,8,9,10,11,12]. Although the nebulization of solutions containing therapeutic nucleic acids has mainly been performed in clinical trials for inhalation due to its ease of introduction [13,14,15], a number of difficulties are associated with its practical use, such as large-scale losses of nucleic acids, long administration times, low pulmonary delivery efficiency, and the cleaning of the nebulizer after each administration. Therefore, the development of inhalable dry powders as alternative formulations for inhalation has been attracting increasing attention because of their practical advantages, such as easy usage, good portability of the device, and high storage stability in the solid state.

The successful development of dry powders for inhalation requires the rational design of particle structures for high aerosol performance. Particles with aerodynamic diameters of 1–5 μm are suitable for pulmonary delivery through inhalation, whereas those with geometric diameters in this range are generally highly adhesive or cohesive, which reduces flowability and dispersibility through inhalation [16]. In most of the commercial dry powder formulations currently available for inhalation, active pharmaceutical ingredients are micronized via milling, followed by loose agglomeration or physical binding to carrier particles with a large diameter (e.g., coarse lactose) to overcome adhesiveness or cohesiveness [17]. However, since therapeutic nucleic acids are pasty or sticky in their solid state, the application of these particle designs is very challenging. As alternative particle designs, other powder-forming techniques, including spray drying (SD) and spray freeze drying (SFD), have been introduced to produce inhalable dry powder particles in which therapeutic nucleic acids are embedded in a matrix constructed with additives [18].

One of the major concerns in the powder formation of nucleic acids is their destabilization. Individual powder-forming techniques involve several physical treatments, such as heating, agitation, atomization, freezing, and drying, some or all of which may reduce the structural and functional integrities of nucleic acids under physical stresses like shear and thermal ones. For the escaped destabilization of nucleic acids through powder formation, non-viral vectors, such as cationic lipids and polycations, were commonly used as powder components for stabilizing effects as well as high transfection efficiency through electrostatic complexing [18]. However, from the viewpoint of safety, it is also desired to stably produce the powders of naked nucleic acids without using vectors.

Regarding attempts at the powder formation of naked nucleic acids, Kuo and Hwang demonstrated that naked pDNA was destabilized through powder formation via SD and SFD [19,20]. We reported that a dry, naked pDNA powder with hyaluronic acid as an additive produced via SFD exhibited higher gene-expressing activity than a similar solution and pDNA polyplex dispersion with polyethylene imine, a gold standard non-viral vector, after its intratracheal administration to mice, despite pDNA being partly destabilized in the powder [12]. On the other hand, naked siRNA was preserved under powder formation via SD and SFD [21,22,23] but was slightly destabilized under powder formation via SD at a higher inlet temperature [24].

Therefore, siRNA appears to be more stable than pDNA under powder formation; however, the different conditions used for powder formation among studies (e.g., the amounts of these nucleic acids and the types of additives included in the powders) may have markedly affected the findings obtained. It is widely known that some additives may act as stabilizers in powder formation for biopharmaceuticals, such as proteins [25]. To the best of our knowledge, a direct comparison of stability between pDNA and siRNA under the same powder-forming conditions has not yet been performed. Detailed and comparative information on the stability of various nucleic acids through each physical treatment, as well as powder formation, is important for their development not only as dry powder formulations for inhalation but also as other formulations, including liquid ones. In addition, for clinical application as high-dose dry powders for inhalation, it is necessary to develop powder-forming techniques that allow a high content of naked nucleic acids to be stably contained in powders; however, excessive content of nucleic acids may reduce the aerosol performance of powders.

Therefore, the present study directly compared the stability of naked pDNA and siRNA under powder formation and various physical treatments through evaluations of structural and functional integrities. Furthermore, we examined the feasibility of successfully producing high-siRNA-dose dry powders for inhalation with good aerosol performance.

## 2. Materials and Methods

### 2.1. Materials

pDNA with the CAG promoter and encoding firefly luciferase (Fluc) (pCAG-Fluc), which was kindly provided by Prof. Dr. K. Kataoka at the Innovation Center of NanoMedicine (iCONM), was used after amplification and purification using the same procedure reported in our previous study [26]. siRNAs specific and not specific to Fluc (siGL3 and siRL (a control siRNA)) were purchased from Samcully PharmPharm. Co., Ltd. (Seoul, Republic of Korea), as described in our previous study [27]. In the powder formation of pDNA and siRNA, D-trehalose dihydrate (Tre: Sigma-Aldrich, St. Louis, MO, USA), D-mannitol (Man: FUJIFILM Wako Pure Chemical Co., Osaka, Japan), inulin (Inu: Sigma-Aldrich, St. Louis, MO, USA), and L-leucine (Leu: FUJIFILM Wako Pure Chemical Co., Osaka, Japan) were used as additives. UltraPure^TM^ DNase/RNase-free distilled water (UPW: Invitrogen Co., Waltham, MA, USA) was used as a solvent. Fluorescein sodium salt (FlNa: Sigma-Aldrich, St. Louis, MO, USA) was adopted as a fluorescent label for the powder used in the evaluation of aerosol performance described below. The other reagents used were of analytical grade.

### 2.2. Cells

Murine colon adenocarcinoma (CT26) cells, kindly provided by Prof. Dr. Y. Takakura, Kyoto University, were employed to assess the functional integrity of pDNA. CT26 cells stably expressing Fluc (CT26/Fluc cells), established in our previous study [27], were used to evaluate the functional integrity of siRNA. These cells were cultivated in a growth medium (RPMI 1640 medium supplemented with 10% heat-inactivated fetal bovine serum, 100 units/mL of penicillin, and 100 μg/mL of streptomycin) at 37 °C in humidified air with 5% CO_2_.

### 2.3. Physical Treatment of pDNA and siRNA

Eighty micrograms each of pDNA and siGL3 were dissolved in 4 mL of UPW. The pDNA and siGL3 solutions were subjected to various physical treatments under the set conditions shown in Table 1. An ultrasonic bath sonicator (UT-205HS, Sharp Corp., Sakai, Japan), a vortex mixer (GENIE2, Electro Scientific Industries, Inc., Beaverton, OR, USA), a two-fluid nozzle (TFN: an inner diameter of 0.4 mm) for a spray dryer (SD-1000, EYELA, Tokyo, Japan), and a freeze dryer (DRC-1100 and FDU-2100, EYELA, Tokyo, Japan) were used for sonification, vortex agitation, atomization, and lyophilization, respectively. At each set time point before and after the treatment (Table 1), the pDNA and siGL3 solutions were partially collected for use in evaluations of structural and functional integrities described below.

### 2.4. Powder Formation of pDNA and siRNA via SD

The compositions of the SD powders produced in the present study are listed in Table 2. All components were dissolved in UPW, adjusting to 5 mg/mL as their total concentration. Using a spray dryer (Nano Spray Dryer B-90, Büchi Labortechnik AG, Flawil, Switzerland), component solutions were flowed into the nozzle with a vibrating mesh of 5.5 µm hole size for atomization, followed by the drying of droplets through water evaporation to obtain SD powders. The inlet temperature and air flow rate were set to 60 or 120 °C and 90 L/min, respectively. The powders produced were collected with a spatula from a collection cylinder. The powder recovery was >20% of the total component mass in the original solution (20 mg).

### 2.5. Powder Formation of pDNA and siRNA via SFD

The compositions of SFD powers produced in the present study are listed in Table 2. All components were dissolved in UPW, adjusting to the individual total concentrations shown in Table 2. To obtain SFD powders, component solutions were treated through the same procedure with liquid nitrogen as described in our previous study [26,27]. Atomization and lyophilization in the procedure were performed under the same conditions as shown in Table 1, using the same atomizer (TFN) and freeze dryer as described in Section 2.3, except that the Penn-Century MicroSprayer^®^ (MS: Model IA-1C and FMJ-250 high-pressure syringe, Penn-Century, Inc., Wyndmoor, PA, USA) was also used for atomization as another manual atomizer. The powders produced were collected with a spatula from a collection container. The powder recovery was >30% of the total component mass in the original solution (12.5–44.3 mg).

### 2.6. Structural Integrities of pDNA and siRNA

The samples collected before and after various physical treatments and the original solutions before powder formation were adjusted with UPW to concentrations of 8.33 μg/mL for pDNA and 750 nM for siGL3. The powders produced were dissolved in UPW at the same concentrations described above. Regarding pDNA, 6 μL of the sample solution (equivalent to 50 ng of pDNA) was loaded on a 0.6% (*w*/*v*) agarose gel, followed by electrophoresis at 100 V and 150 mA for 2 h in Tris-acetate-EDTA running buffer. Concerning siGL3, 10 μL of the sample solution (equivalent to 7.5 pmol of siGL3) was loaded on a 15% (*w*/*v*) polyacrylamide gel, followed by electrophoresis at 250 V and 30 mA for 45 min in Tris-borate-EDTA running buffer. As standards for pDNA, fresh pDNA, digested pDNA (by Hind III), and a molecular size marker for pDNA (Loading Quick^®^ λ/Hind III digest, Toyobo Co., Ltd., Osaka, Japan) were similarly loaded for electrophoresis, as were fresh siGL3 and a molecular size marker for siRNA (N2101S, New England BioLabs, Beverly, MA, USA) as standards for siGL3. After electrophoresis, these gels were soaked in 0.5 μg/mL ethidium bromide solution to observe bands corresponding to pDNA and siGL3 with a fluorescence image analyzer (Typhoon^TM^ FLA 9000, GE Healthcare, Piscataway, NJ, USA). Band intensities for pDNA and siRNA were measured using dedicated software (ImageQuant TL (version 8.1), GE Healthcare, Piscataway, NJ, USA). Linear correlations with band intensities for the supercoiled and open-circular forms of pDNA and intact siGL3 were confirmed in the amount ranges of 3.1–50 ng for pDNA and 0.47–7.5 pmol for siGL3 (correlation coefficients of >0.99).

### 2.7. Functional Integrities of pDNA and siRNA

The samples collected at the final time points after various physical treatments were adjusted with Opti-MEM^®^ (Thermo Fisher Scientific, Waltham, MA, USA) to concentrations of 32 μg/mL for pDNA and 600 nM for siRNA. The powders produced were dissolved in Opti-MEM^®^ at the same concentrations described above. Lipofectamine^TM^ 2000 (Invitrogen Co., Waltham, MA, USA), a transfection reagent, was added to these solutions at ratios of 2.5 μL/μg of pDNA and 0.1 μL/pmol of siRNA, adjusted to 16 μg/mL for pDNA and 75, 150, or 300 nM for siRNA in transfection media. As standards, fresh pDNA and siGL3 were handled under the same procedure as that described above.

Regarding pDNA transfection, CT26 cells were seeded on a 24-well microplate at a concentration of 60,000 cells/600 μL/well. After an incubation for 24 h, the growth medium was replaced with 500 μL of Opti-MEM^®^. One hundred microliters of the transfection medium for pDNA was added to each well (final exposure concentration of 2.67 μg/mL as pDNA), followed by an incubation for 4 h. The transfection medium was then replaced with 600 μL of the fresh growth medium, followed by further incubation for 44 h. After the incubation, the growth medium was replaced with a lysis buffer (0.05% Triton X-100, 2 mM of EDTA, and 0.1 M of Tris, pH 7.8) in each well to lyse the attached cells. To achieve complete cell lysis, the lysate was treated with three cycles of freezing and thawing, followed by centrifugation at 13,000× *g* at 4 °C for 7 min to collect the supernatant. Regarding the supernatant, luminescence intensity corresponding to Fluc expression was measured with the luciferase reporter assay with a dedicated kit (Picagene^®^ Luminescence Kit, Toyo Ink Co., Ltd., Tokyo, Japan) using a luminometer (Lumat LB9507, Berthold Technologies, Bad Wildbad, Germany), and the protein concentration was assessed with the Bradford assay with Coomassie brilliant blue G 250 (Sigma-Aldrich, St. Louis, MO, USA) using a microplate reader (Model 550, Bio-Rad Laboratories, Inc., Hercules, CA, USA). The Fluc expression was defined using the following equation:

Fluc expression (RLU/mg protein) = LI/C,(1)

where LI and C are the luminescence intensity and protein concentration, respectively, in the supernatant.

Concerning siRNA transfection, CT26/Fluc cells were seeded on a 96-well microplate at a concentration of 10,000 cells/100 μL/well. After an incubation for 24 h, the growth medium was replaced with 100 μL of Opti-MEM^®^. Fifty microliters of the transfection solution for siRNA was added to each well (final exposure concentration of 25, 50, or 100 nM as siRNA), followed by an incubation for 4 h. The transfection medium was then replaced with 100 μL of the fresh growth medium, followed by further incubation for 44 h. A total of 10 μL of alamarBlue^®^ reagent (Thermo Fisher Scientific, Waltham, MA, USA) for the cell viability assay and 10 μL of 150 μg/mL of luciferin solution (in phosphate-buffered saline (PBS), Promega, Madison, WI, USA) for the luciferase reporter assay were added to each well 40 and 43.5 h, respectively, after the initiation of the incubation for 44 h. After finishing the incubation, fluorescence generated through the reaction with alamarBlue^®^ reagent (λ_ex_: 465 nm; λ_em_: 600 nm) and luminescence corresponding to Fluc expression were detected using an in vivo imaging system (IVIS-SPECTRUM, PerkinElmer, Waltham, MA, USA) to measure their intensities in each well. The Fluc expression was defined using the following equation:

Fluc expression (% of Control) = (LI/FI)/(LI_non_/FI_non_) × 100, (2)

where LI and LI_non_ are the luminescence intensities in wells with cells transfected with siRNA and with non-transfected cells, respectively, while FI and FI_non_ are the fluorescence intensities for wells with cells transfected with siRNA and with non-transfected cells, respectively.

### 2.8. Powder Particle Morphology

The powders produced were dispersed with a simple apparatus for powder dispersion [12] to deposit them on a specimen stub with double-sided carbon tape. The deposited powders were coated with platinum using a sputter coater (JFC-1600, JEOL, Tokyo, Japan), followed by morphological observations using a scanning electron microscope (JSM-6060, JEOL, Tokyo, Japan).

### 2.9. Powder Aerosol Performance

The aerosol performance of the powders produced was evaluated with the same procedure described in our previous study [26,27] using an 8-stage Andersen cascade impactor (ACI: AN-200, Sibata Scientific Technology, Saitama, Japan). A device for oral powder inhalation (Jethaler^®^ reverse type, Tokiko System Solutions, Ltd., Kawasaki, Japan), containing 2 mg of the powder loaded into a No. 2 hydroxypropyl methylcellulose capsule (Shionogi Qualicaps Co., Ltd., Nara, Japan), was connected to the ACI via an induction port (throat), followed by inspiration for 5 s with a suction pump. The inspiratory flow rate was set to 28.3 L/min, which is equivalent to the average value of the peak inspiratory flow rate for the device reached after inhalation in healthy volunteers [28]. After inspiration, the powder deposited in each part was collected by dissolving in 10 mL of PBS. The fluorescence intensity of FlNa (λ_ex_: 490 nm; λ_em_: 515 nm) in the collected powder solution was measured using a fluorescence microplate reader (SpectraMax Gemini EM, Molecular Devices, Sunnyvale, CA, USA) to quantify powder deposition in each part. As aerosol performance indices, the emitted fraction (EF), fine particle fraction (FPF), and ultrafine particle fraction (UPF) were calculated from the following equations:

EF (%) = M_emitted_/M_total_ × 100, (3)

FPF (%) = M_<4.7 μm_/M_total_ × 100, (4)

UPF (%) = M_<2.1 μm_/M_total_ × 100, (5)

where M_total_, M_emitted_, M_<4.7 μm_, and M_<2.1 μm_ are the total mass collected, the mass collected from the throat and the lower parts, the mass collected from stage 3 and the lower parts (equivalent to that of the powder with an aerodynamic diameter < 4.7 μm), and the mass collected from stage 5 and the lower parts (equivalent to that of the powder with an aerodynamic diameter < 2.1 μm), respectively. Based on the deposition pattern in the ACI, the mass median aerodynamic diameter (MMAD) of the powder was measured using dedicated software (aerosol particle density analysis system (version 3.0E), Sibata Scientific Technology, Saitama, Japan).

### 2.10. Statistical Analysis

Statistical comparisons in the evaluation of functional integrity were performed with a one-way analysis of variance (ANOVA), followed by Dunnett’s test, while those in the evaluation of aerosol performance were conducted with an ANOVA, followed by Fisher’s least significant difference test. *p* < 0.05 was considered to be significant.

## 3. Results

### 3.1. Structural Integrities of pDNA and siRNA after Various Physical Treatments

**Figure 1:**
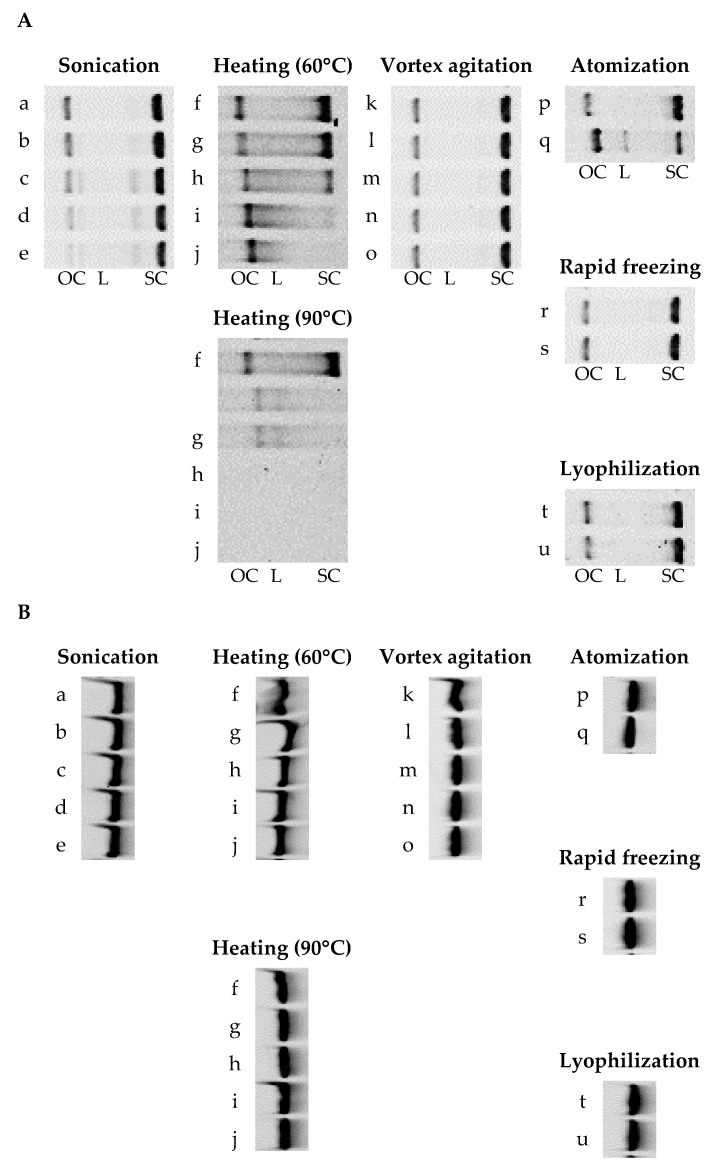
Gel electrophoresis images of (**A**) pDNA and (**B**) siRNA after various physical treatments. Sonication: (a) 0 min, (b) 1 min, (c) 5 min, (d) 10 min, and (e) 30 min. Heating: (f) 0 min, (g) 5 min, (h) 15 min, (i) 30 min, and (j) 60 min. Vortex agitation: (k) 0 min, (l) 0.5 min, (m) 1 min, (n) 3 min, and (o) 5 min. Atomization: just (p) before and (q) after. Rapid freezing: just (r) before and (s) after. Lyophilization: just (t) before and (u) after. OC: open-circular, L: linear, and SC: supercoiled.

Figure 1 shows the gel electrophoresis images of pDNA and siRNA after various physical treatments. As pDNA is continuously damaged, the supercoiled form is converted to the open-circular form, subsequently generating the linear form with lower biological activity. The positions of bands corresponding to the supercoiled, open-circular, and linear forms of pDNA were identified from the results for fresh pDNA, digested pDNA, and the molecular size marker for pDNA, as was that corresponding to intact siGL3 from the results for the molecular size marker for siRNA (Figure S1). Band intensities for pDNA (its supercoiled and open-circular forms) and siGL3 were analyzed from these images, followed by calculations of relative band intensities for samples 0 min after or just before the treatment (Table 3).

The treatment of pDNA with sonication or heating at 90 °C decreased relative band intensities for its supercoiled and open-circular forms to less than 70 and 35%, respectively, indicating that the structural integrity of pDNA was reduced by these physical treatments. The treatment of pDNA with heating at 60 °C or atomization decreased the relative band intensity for its supercoiled form to less than 50% but increased that for its open-circular form to more than 150%. On the other hand, after the treatment of pDNA with vortex agitation, rapid freezing, or lyophilization, relative band intensities for both its supercoiled and open-circular forms were 80–110%. A band corresponding to the linear form of pDNA was only observed after treatment with atomization. In contrast, the relative band intensity for siGL3 was more than 80% after all the physical treatments investigated, demonstrating the higher stability of siRNA than that of pDNA under physical treatments.

### 3.2. Functional Integrities of pDNA and siRNA after Various Physical Treatments

**Figure 2:**
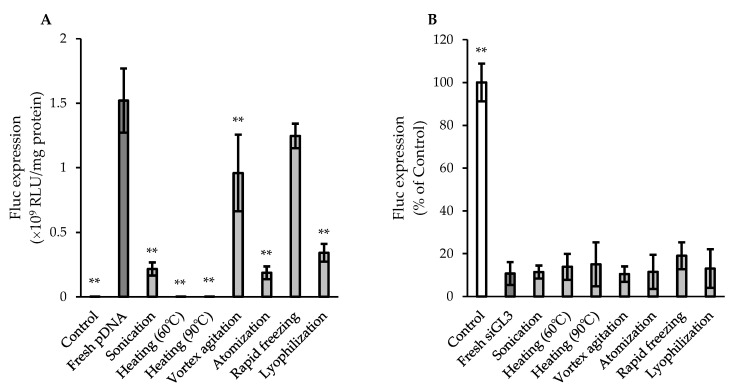
In vitro (**A**) gene-expressing activity of pDNA and (**B**) gene-silencing activity of siRNA after various physical treatments. The samples collected at the final time points after individual physical treatments were used for the evaluation. The final exposure concentration of siRNA in (**B**) was 100 nM. Each value represents the mean ± S.D. (*n* = 3–4). Significant from (**A**) fresh pDNA (** *p* < 0.01) and (**B**) fresh siGL3 (** *p* < 0.01).

Figure 2 shows the gene-expressing activity of pDNA and the gene-silencing activity of siRNA after various physical treatments, which were evaluated based on the Fluc expression in cultured cells (CT26 cells for the pDNA evaluation and CT26/Fluc cells for the siRNA evaluation). Fluc expression for pDNA treated with sonication, heating at 60 and 90 °C, atomization, or lyophilization was less than 25% of that for fresh pDNA, indicating a significant reduction in functional integrity by these physical treatments. On the other hand, the Fluc expression for pDNA treated with vortex agitation or rapid freezing was more sufficiently preserved (approximately 63 and 82% of fresh pDNA in vortex agitation and rapid freezing, respectively), whereas a significant difference was observed between pDNA treated with vortex agitation and fresh pDNA. In contrast, siGL3 after all the physical treatments investigated exhibited the almost equivalent suppression of Fluc expression to fresh siGL3 (10–20% of Control), demonstrating that siRNA was more stable than pDNA under physical treatments, similar to the results obtained in the evaluation of structural integrity (Figure 1 and Table 3).

### 3.3. Structural Integrities of pDNA and siRNA after Powder Formation

**Figure 3:**
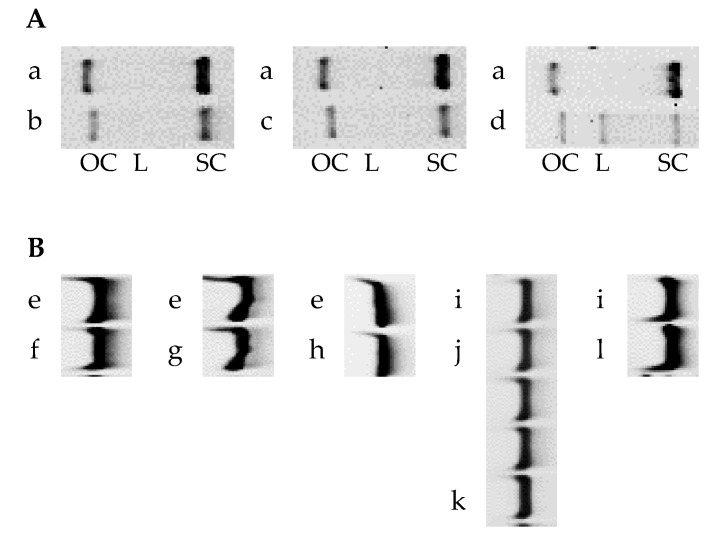
Gel electrophoresis images of (**A**) pDNA and (**B**) siRNA after powder formation. (a) The original pDNA solution before powder formation and the powders: (b) pDNA SD#1, (c) pDNA SD#2, and (d) pDNA SFD. (e) The original solution before powder formation and the powders: (f) siGL3 SD#1, (g) siGL3 SD#2, and (h) siGL3 SFD#1. (i) Fresh siGL3 and the powders: (j) siGL3 SFD#2, (k) siGL3 SFD#3, and (l) siGL3 SFD#4. OC: open-circular, L: linear, and SC: supercoiled.

Figure 3 shows the gel electrophoresis images for pDNA and siRNA after powder formation via SD and SFD under different production conditions. In the same manner, as described in Section 3.1, the positions of bands corresponding to pDNA and siGL3 were identified from the original images (Figure S2), followed by an analysis of relative band intensities to the original solutions before powder formation or fresh siGL3 (Table 4).

Regarding powder formation with Tre and Leu as additives, relative band intensities for the open-circular form of pDNA in the SD and SFD powders (pDNA SD#1, pDNA SD#2, and pDNA SFD) were almost equivalently low (40–50%), whereas those for the supercoiled form were low in the following order: pDNA SFD (25%) < pDNA SD#2 (35%) < pDNA SD#1 (43%). A band corresponding to the linear form was only observed in pDNA SFD. These results clearly indicated a partial reduction in the structural integrity of pDNA under powder formation. In contrast, although the relative band intensity for siGL3 in the SFD powder (siGL3 SFD#1) was slightly lower than those in the SD powders (siGL3 SD#1 and siGL3 SD#2), these intensities were more than 85%, demonstrating that siRNA was much more stable than pDNA under powder formation.

The evaluation of the structural integrity of siRNA based on gel electrophoresis imaging was performed on the other SFD powders (siGL3 SFD#2, siGL3 SFD#3, and siGL3 SFD#4) produced with two types of atomizers (TFN and MS), containing a larger amount of siGL3 (3 or 12% of the powder) and different additives (Man, Leu, and Inu). Consequently, relative band intensities for siGL3 were almost 100% (95–130%) in all the SFD powders investigated, irrespective of the various production conditions described above.

### 3.4. Functional Integrities of pDNA and siRNA after Powder Formation

**Figure 4:**
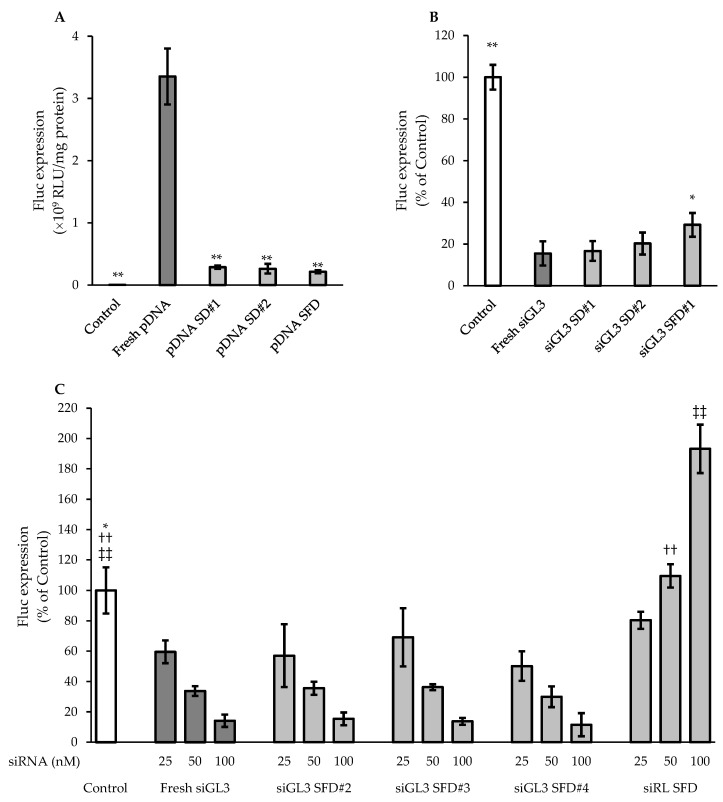
In vitro (**A**) gene-expressing activity of pDNA and (**B**,**C**) gene-silencing activity of siRNA after powder formation. The final exposure concentration of siRNA in (**B**) was 100 nM. Each value represents the mean ± S.D. (*n* = 3–4). Significant difference from (**A**) fresh pDNA (** *p* < 0.01), (**B**) fresh siGL3 (** *p* < 0.01; * *p* < 0.05), and (**C**) fresh siGL3 (* *p* < 0.05 at 25 nM as siRNA; ^††^ *p* < 0.01 at 50 nM as siRNA; and ^‡‡^ *p* < 0.01 at 100 nM as siRNA).

Figure 4 shows the gene-expressing activity of pDNA and the gene-silencing activity of siRNA after powder formation via SD and SFD under different production conditions, which were evaluated in the same manner as described in Section 3.2.

Regarding powder formation with Tre and Leu as additives, the Fluc expressions for pDNA in pDNA SD#1, pDNA SD#2, and pDNA SFD were 8.5, 7.8, and 6.4%, respectively, of that for fresh pDNA, indicating the significantly reduced functional integrity of pDNA after powder formation. In contrast, siGL3 in all SD and SFD powders (siGL3 SD#1, siGL3 SD#2, and siGL3 SFD#1) exhibited the almost equivalent suppression of Fluc expression to fresh siGL3 (15–30% of Control); however, efficacy was slightly lower in siGL3 SFD#1 than in siGL3 SD#1 and siGL3 SD#2. These results demonstrated that siRNA was more stable than pDNA under powder formation, similar to the results obtained in the evaluation of structural integrity (Figure 3 and Table 4).

Furthermore, the gene-silencing activity of siRNA in the other SFD powders, which were produced using two types of atomizers (TFN and MS) and containing a larger amount of siRNA (3 or 12% of the powder) and different additives (Man, Leu, and Inu), was evaluated at several siRNA concentrations. Irrespective of various production conditions, siGL3 in all the powders (siGL3 SFD#2, siGL3 SFD#3, and siGL3 SFD#4) showed its concentration-dependent suppression of the Fluc expression, which was almost equivalent to fresh siGL3 at each set siRNA concentration. On the other hand, suppression was not observed in siRL SFD, which was produced with siRNA not specific to Fluc under the same conditions set for siGL3 SFD#4, confirming the sequence-specific gene-silencing effects of siRNA. On the contrary, siRL in the SFD powder increased Fluc expression by about 200% compared to Control at 100 nM as siRNA, indicating its unspecific effect.

### 3.5. Particle Morphologies of SD and SFD Powders Produced

**Figure 5:**
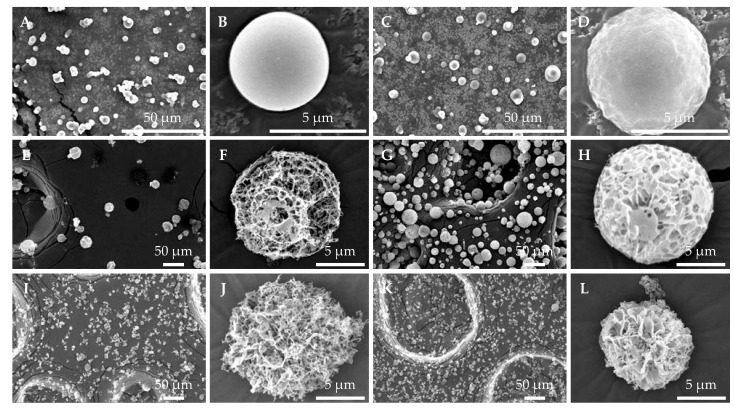
Scanning electron micrographs of SD and SFD powders produced: (**A**,**B**) pDNA SD#1, (**C**,**D**) siGL3 SD#1, (**E**,**F**) siGL3 SFD#2, (**G**,**H**) siGL3 SFD#3, (**I**,**J**) siGL3 SFD#4, and (**K**,**L**) siRL SFD. (**A**,**C**,**E**,**G**,**I**,**K**) low magnification and (**B**,**D**,**F**,**H**,**J**,**L**) high magnification.

Representative SEM images of the SD and SFD powders produced are shown in Figure 5. The SD powders with Tre and Leu as additives (pDNA SD#1 and siGL3 SD#1) had spherical structures with diameters of 5–10 μm and relatively smooth surfaces. On the other hand, the structures of the SFD powders with the same additives (pDNA SFD and siGL3 SFD#1) were not distinctly observed in SEM images, which may be attributed to their low stability through sputter coating with platinum. The other SFD powders had highly porous and spherical structures, characterized by the SFD process, irrespective of their compositions. The SFD powders produced with MS (siGL3 SFD#2 and siGL3 SFD#3) had diameters of 10–50 μm, whereas those with TFN (siGL3 SFD#4 and siRL SFD) had diameters of 5–10 μm.

### 3.6. Aerosol Performance of SFD Powder for High-Dose Naked siRNA

**Figure 6:**
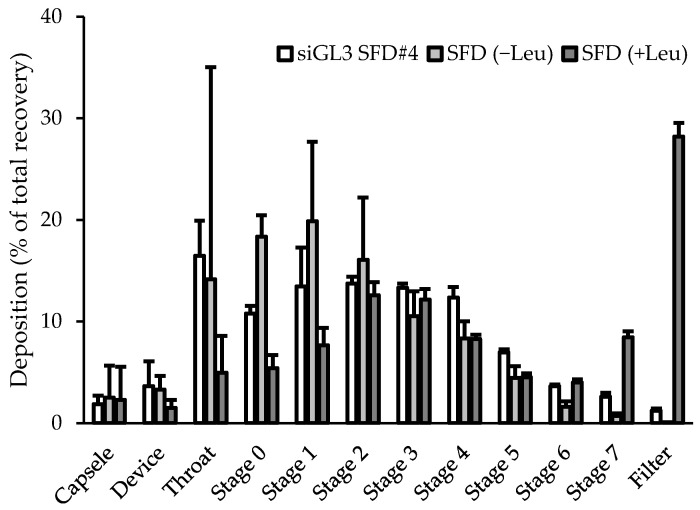
Aerosol deposition patterns of SFD powders in ACI. Each value represents the mean + S.D. (*n* = 3).

The aerosol performance of siGL3 SFD#4, which had the highest content of siGL3 (12% of the powder), was assessed using the ACI for comparisons with those of SFD (−Leu) and SFD (+Leu) without siGL3. In their deposition patterns (Figure 6), siGL3 SFD#4 showed small deposition in the capsule and device, similar to SFD (−Leu) and SFD (+Leu). In the lower stages, SFD#4 showed a larger deposition than SFD (−Leu) but a smaller deposition than SFD (+Leu). Regarding the aerosol performance indices analyzed from their deposition patterns (Table 5), siGL3 SFD#4 achieved an EF of ~95%, FPF of ~40%, UPF of ~14%, and MMAD of ~3.8 μm, demonstrating excellent aerosol performance suitable for pulmonary delivery through inhalation. These values were between those for SFD (−Leu) and SFD (+Leu), indicating that the aerosol performance of siGL3 SFD#4 was improved with the addition of Leu; however, the extent of this improvement was reduced by the high content of siGL3.

## 4. Discussion

The present study demonstrated that siRNA was significantly more stable than pDNA under both physical treatments and powder formation, as shown by the evaluations of structural and functional integrities. A correlation was observed between the reduction in structural integrity (corresponding to the supercoiled form in the case of pDNA) and decreases in the functional integrities of pDNA and siRNA after physical treatments and powder formation. Functional integrity exhibited a more significant decline than structural integrity in both physical treatments and powder formation. These results suggest the necessity of evaluating functional integrity for more accurate investigations on the stability of pDNA or siRNA; structural integrity has already been examined in detail [19,20,29,30,31].

Among the physical treatments investigated, heating induced the largest reduction in the functional integrity of pDNA and the structural integrity of its supercoiled form (Figure 2 and Table 3). Yan and Iwasaki detected the temperature-dependent structural shift in pDNA using atomic force microscopy and showed that it had a globule structure inside the DNA chain at temperatures higher than 50 °C through partial denaturation and a granular structure at those higher than 80 °C due to complete denaturation and the collapse of single strands [32]. The temperature-dependent shift may support the marked reductions observed in the structural and functional integrities of pDNA described above and explain the different results obtained on the relative band intensity of its open-circular form between 60 and 90 °C in the present study (Table 3). Sonication and atomization significantly decreased the functional integrity of pDNA and the structural integrity of its supercoiled form, which may be due to the high shear stresses caused by these treatments [20,31,33]. Vortex agitation, rapid freezing, and lyophilization did not reduce the structural integrity of pDNA, while only lyophilization significantly decreased its functional integrity, suggesting that lyophilization induced a structural change in pDNA that was not detected via electrophoresis. The decrease observed in the functional integrity of pDNA after lyophilization is consistent with previous findings [34]. Regarding the powder-forming techniques introduced in the present study, SD involves two physical treatments: atomization and heating, while SFD comprises atomization, rapid freezing, and lyophilization. In comparison with the reductions observed in the structural and functional integrities of pDNA after heating, those detected after powder formation via SD were modest, which may be attributed to lower thermal stress due to the short exposure time at a high inlet temperature and an endothermal reaction through evaporation. The SFD powder (pDNA SFD) showed slightly larger reductions in the structural and functional integrities of pDNA or siRNA than the SD powders (pDNA SD#1 and pDNA SD#2), which may have been due to the higher physical stresses in SFD through many physical treatments, higher-shear atomization with TFN, and impaction with liquid nitrogen. In contrast, the structural and functional integrities of siRNA were preserved after all the physical treatments and powder formation. The high stability of siRNA observed in the present study is consistent with previous findings [21,22,23,24,35,36]. The physical stresses caused by SD in the present study may have been weaker than those reported by Wu et al., who showed that the functional integrity of siRNA decreased in SD powders with increases in the inlet temperature (80 to 200 °C) and atomizing airflow (473 to 742 L/h) [24]. Although the reasons for the high stability of siRNA remain unclear, a decrease in the number of base pairs in pDNA may reduce the shear force loaded on it [29,30]. Therefore, fewer base pairs in siRNA may be a factor contributing to its high stability.

In the first powder formation in the present study to compare the stability of pDNA and siRNA, Tre was used as the main additive because it has been widely accepted as one of the most effective stabilizers for the powder formation of proteins and liposomes [25,37]. Water replacement and vitrification have been proposed as the mechanisms responsible for its stabilizing effects [25,37]. However, Tre did not markedly contribute to the stability of pDNA and siRNA under powder formation in the present study because no significant differences were observed between physical treatment testing without Tre (Figure 1 and Figure 2 and Table 3) and powder-forming testing with Tre (Figure 3 and Figure 4 and Table 4). On the other hand, the SFD powders with Tre (pDNA SFD and siGL3 SFD#1) were estimated to have low stability based on the failure in the distinct observations of their structures via SEM. Although Tre is stable in the dihydrate crystal form, pDNA SFD and siGL3 SFD#1 were speculated to have highly porous structures with large surface areas, similar to the other SFD powders (Figure 5), as well as the anhydrous form of Tre with low crystallinity [37], which may consequently have lower stability against moisture and the other stimulants. In comparison with these SFD powders, the SD powders with Tre (pDNA SD#1, pDNA SD#2, siGL3 SD#1, and siGL3 SD#2) appeared to be stable but unsuitable for inhalation due to their high cohesiveness and poor dispersibility. According to these results, the further development of inhalable dry powders stably carrying a higher content of naked siRNA was proceeded by SFD with other main additives (Man and Inu). The SFD powders with Man and Inu (siGL3 SFD#2, siGL3 SFD#3, and siGL3 SFD#4) stably carried naked siRNA at a higher content (3 or 12% of the powder) irrespective of the different atomizers used (Figure 3 and Figure 4 and Table 4).

In the process of SFD, frozen droplets containing powder components are instantly generated in liquid nitrogen, and this is followed by the removal of the frozen solvent (e.g., ice) in the droplets through sublimation without the movement of powder components to form highly porous dry powder particles. Therefore, SFD powders are speculated to have similar diameters to droplets after atomization. MS, the atomizer used to produce siGL3 SFD#2 and siGL3 SFD#3, generates droplets of a diameter of 16–22 μm (described in the product information), which is close to the diameters of these SFD powders (Figure 5). The smaller diameters of siGL3 SFD#4 and siRL SFD (Figure 5) may be attributed to the smaller diameters of droplets generated with TFN, the atomizer used to produce the powders. In the process of SD, the diameter of droplets containing powder components decreases in heated gas flow due to the removal of the solvent in the droplets through evaporation, thereby forming smaller dry powder particles than the initial droplets. The decreased diameter of droplets may be the main reason why the diameters of pDNA SD#1 and siGL3 SD#1 were markedly smaller than those of all the SFD powders investigated, taking into consideration that the vibrating mesh atomizer used to produce pDNA SD#1 and siGL3 SD#1 generates droplets with a similar diameter to MS (approximately 15 μm, described in the product information).

SFD powders have great potential in clinical applications as inhaled drugs due to their highly porous structures with low densities, allowing powders with aerodynamic diameters of 1–5 μm (suitable for pulmonary delivery through inhalation) to achieve larger geometric diameters with low cohesiveness and high dispersibility [38]. In our previous study, the addition of Leu to an SFD powder markedly improved its aerosol performance due to a reduced cohesive force and increased surface potential, leading to enhanced dispersibility [39]. Leu is a common additive in SD powders for the same purpose [40]. Therefore, it was used in the present study to enhance the dispersibility of the SD and SFD powders. siGL3 SFD#4, the SFD powder with Leu and a high content of siGL3 (12% of the powder), showed similar high aerosol performance with an FPF of ~40% (Table 5), equivalent to or higher than FPFs in commercial dry powder products for inhalation [41]. Nevertheless, it is important to note that further increases in the siRNA content may reduce the aerosol performance of the SFD powder. To the best of our knowledge, the siRNA content of 12% in the powder set in the present study is the highest reported to date. Chow et al. found that the addition of Leu at 50% was necessary to achieve an FPF of >30% in the SD powder with 2% siRNA [21]. However, the present study showed that the addition of Leu at 5% achieved the objective FPF in siGL3 SFD#4, the SFD powder with 12% siRNA (Table 5). The ideal Leu content for improving the aerosol performance of SD and SFD powders has been verified in other studies; we previously identified 5% Leu as optimal for an SFD powder [39], whereas other studies added ≥20% Leu to SD powders [40]. A lower Leu content in SFD powders is advantageous because it minimizes possible adverse effects caused by hydrophobic Leu; however, the underlying mechanisms remain unclear.

## 5. Conclusions

Regarding the stability of naked pDNA and siRNA under various physical treatments, pDNA is strongly destabilized via sonication, heating, atomization, and lyophilization, whereas it may be almost perfectly and partly preserved after rapid freezing and vortex agitation, respectively. On the other hand, siRNA may be almost perfectly preserved after all the physical treatments described above. Similarly, siRNA is more stable than pDNA under powder formation via SD and SFD, regardless of the composition of additives. A highly porous dry powder produced via SFD with Man and Leu as additives may stably carry naked siRNA at a high content (12% of the powder), showing excellent aerosol performance. The present results provide important information for the future development of various naked nucleic acids as dry powder formulations for inhalation.

## Figures and Tables

**Table 1:** Conditions and sampling times set for each physical treatment.

Treatment	Condition	Sampling Time
Sonication	35 kHz, 100 W	0, 1, 5, 10, and 30 * min after treatment
Heating	60 or 90 °C	0, 5, 15, 30, and 60 * min after treatment
Vortex agitation	Strength of vortex 10 in GENIE2	0, 0.5, 1, 3, and 5 * min after treatment
Atomization	150 kPa of atomizing air pressure	Just before and after * treatment
Rapid freezing	Soaking into liquid nitrogen	Just before and after * treatment
Lyophilization	Initial/final shelf temperature: −40/10 °C Sublimation time: 24 h Final vacuum pressure: 1 Pa or less	Just before and after * treatment

* Samples collected at these time points were used in the evaluation of functional integrity.

**Table 2:** Atomization conditions for SD and SFD powders produced in the present study.

Formulation Name	Composition (*w*/*w*%)	Total Component Concentration (mg/mL)	Specification
pDNA SD#1	pCAG-Luc/Tre/Leu = 0.4/94.6/5	5	Inlet temperature: 60 °C
pDNA SD#2	pCAG-Luc/Tre/Leu = 0.4/94.6/5	5	Inlet temperature: 120 °C
siGL3 SD#1	siGL3/Tre/Leu = 0.4/94.6/5	5	Inlet temperature: 60 °C
siGL3 SD#2	siGL3/Tre/Leu = 0.4/94.6/5	5	Inlet temperature: 120 °C
pDNA SFD	pCAG-Luc/Tre/Leu = 0.4/94.6/5	5	Atomizer: TFN
siGL3 SFD#1	siGL3/Tre/Leu = 0.4/94.6/5	5	Atomizer: TFN
siGL3 SFD#2	siGL3/Man/Leu = 3/77/20	25	Atomizer: MS
siGL3 SFD#3	siGL3/Inu = 3/97	100	Atomizer: MS
siGL3 SFD#4	siGL3/Man/Leu/FlNa = 12/82/5/1	25	Atomizer: TFN
siRL SFD	siRL/Man/Leu = 12/83/5	25	Atomizer: TFN
SFD (−Leu)	Man/FlNa = 99/1	25	Atomizer: TFN
SFD (+Leu)	Man/Leu/FlNa = 94/5/1	25	Atomizer: TFN

**Table 3:** Relative band intensities for pDNA and siRNA after various physical treatments in gel electrophoresis images.

Treatment	Sampling Time	Relative Band Intensity for pDNA (%)		Relative Band Intensity for siGL3 (%)
		OC	SC	
Sonication	1 min	104	103	102
	5 min	80.4	89.4	101
	10 min	58.0	79.1	102
	30 min	32.7	68.9	101
Heating (60 °C)	5 min	88.9	70.1	101
	15 min	104	32.1	101
	30 min	165	13.6	101
	60 min	166	7.69	101
Heating (90 °C)	5 min	62.2	13.3	98.8
	15 min	9.54	8.42	98.2
	30 min	3.95	6.25	97.8
	60 min	3.74	5.15	93.8
Vortex agitation	0.5 min	88.9	97.7	83.3
	1 min	83.2	93.3	84.1
	3 min	90.0	94.1	92.4
	5 min	95.3	97.7	90.2
Atomization	Just after	209	47.6	90.1
Rapid freezing	Just after	124	107	107
Lyophilization	Just after	88.2	95.8	102

OC: open-circular and SC: supercoiled.

**Table 4:** Relative band intensities for (A) pDNA and (B) siRNA after powder formation in gel electrophoresis images.

(A)		
Formulation Name	Relative Band Intensity (%)	
	OC	SC
pDNA SD#1	44.7	43.2
pDNA SD#2	47.2	34.8
pDNA SFD	45.3	25.4
**(B)**		
**Formulation Name**	**Relative Band Intensity (%)**	
siGL3 SD#1	99.7	
siGL3 SD#2	99.9	
siGL3 SFD#1	86.3	
siGL3 SFD#2	98.7	
siGL3 SFD#3	126	
siGL3 SFD#4	118	

OC: open-circular and SC: supercoiled.

**Table 5:** Various aerosol performance indices for SFD powders.

Formulation Name	EF (%)	FPF (%)	UPF (%)	MMAD (μm)
siGL3 SFD#4	94.5 ± 2.8	40.1 ± 0.8 **^,††^	14.4 ± 0.4 **^,††^	3.80 ± 0.20 **^,††^
SFD (−Leu)	94.2 ± 4.4	25.7 ± 5.7	6.8 ± 2.0	5.87 ± 0.42
SFD (+Leu)	96.2 ± 4.0	65.6 ± 3.3 **	45.2 ± 2.4 **	0.95 ± 0.11 **

Each value represents the mean ± S.D. (*n* = 3). Significant difference from SFD (−Leu) (** *p* < 0.01) and with SFD (+Leu) (^††^ *p* < 0.01).

## Data Availability

Data are contained within the article and supplementary materials.

## Supplementary Materials

The following supporting information can be downloaded at https://www.mdpi.com/article/10.3390/pharmaceutics15122786/s1, Figure S1: Gel electrophoresis images for identifying (A) pDNA and (B) siRNA structures in Figure 1; Figure S2: Gel electrophoresis images for identifying (A) pDNA and (B) siRNA structures in Figure 3.
