# Supplementary material for: Stability of Naked Nucleic Acids under Physical Treatment and Powder Formation: Suitability for Development as Dry Powder Formulations for Inhalation
Source: Pharmaceutics. 2023 Dec 16;15(12):2786. doi: 10.3390/pharmaceutics15122786 (PMC10747740; doi:10.3390/pharmaceutics15122786)
Supplement: Supplementary file 1 [file pharmaceutics-15-02786-s001.zip › Captions for Supplementary Figures (Pharmaceutics, 20231208).pdf]

Captions for Supplementary Figures

Figure S1. Gel electrophoresis images for identifying (A) pDNA and (B) siRNA structures in Figure 1: (a) molecular size marker for pDNA (4361, 6557, and 9416 bp), (b) fresh pDNA, (c) digested pDNA, and (d) molecular size marker for siRNA (17, 21, and 25 bp). Dotted squares represent the regions shown in Figure 1. OC: open-circular, L: linear, SC: supercoiled, and bp: base pairs.

Figure S2. Gel electrophoresis images for identifying (A) pDNA and (B) siRNA structures in Figure 3: (a) molecular size marker for pDNA (4361, 6557, and 9416 bp), (b) fresh pDNA, (c) digested pDNA, and (d) molecular size marker for siRNA (17, 21, and 25 bp). Dotted squares represent the regions shown in Figure 3. OC: open-circular, L: linear, SC: supercoiled, and bp: base pairs.
