# Supplementary figures and images for: Stability of Naked Nucleic Acids under Physical Treatment and Powder Formation: Suitability for Development as Dry Powder Formulations for Inhalation
Source: Pharmaceutics. 2023 Dec 16;15(12):2786. doi: 10.3390/pharmaceutics15122786 (PMC10747740; doi:10.3390/pharmaceutics15122786)

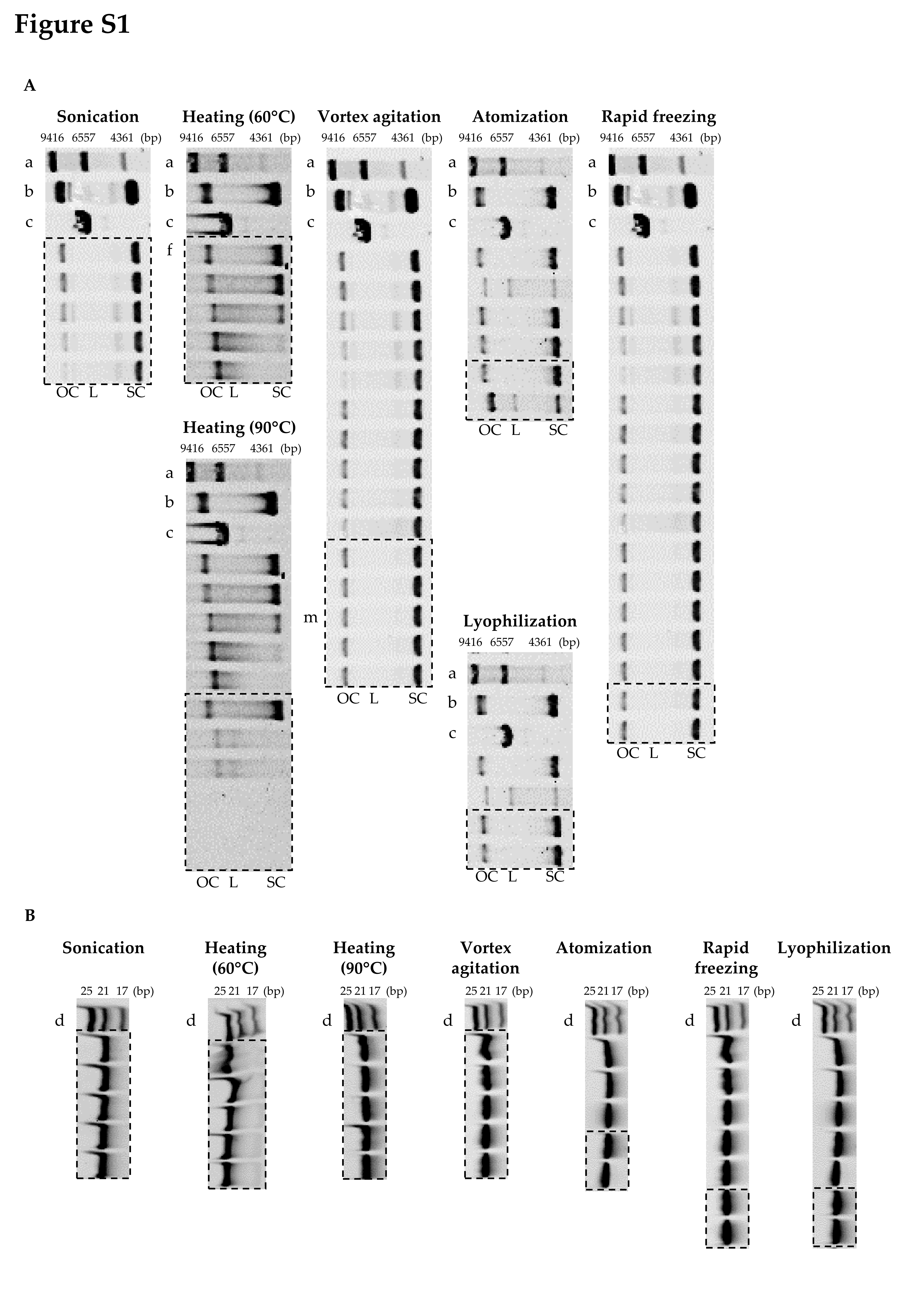

Supplement: Supplementary file 1 [file pharmaceutics-15-02786-s001.zip › Figure S1.tif]

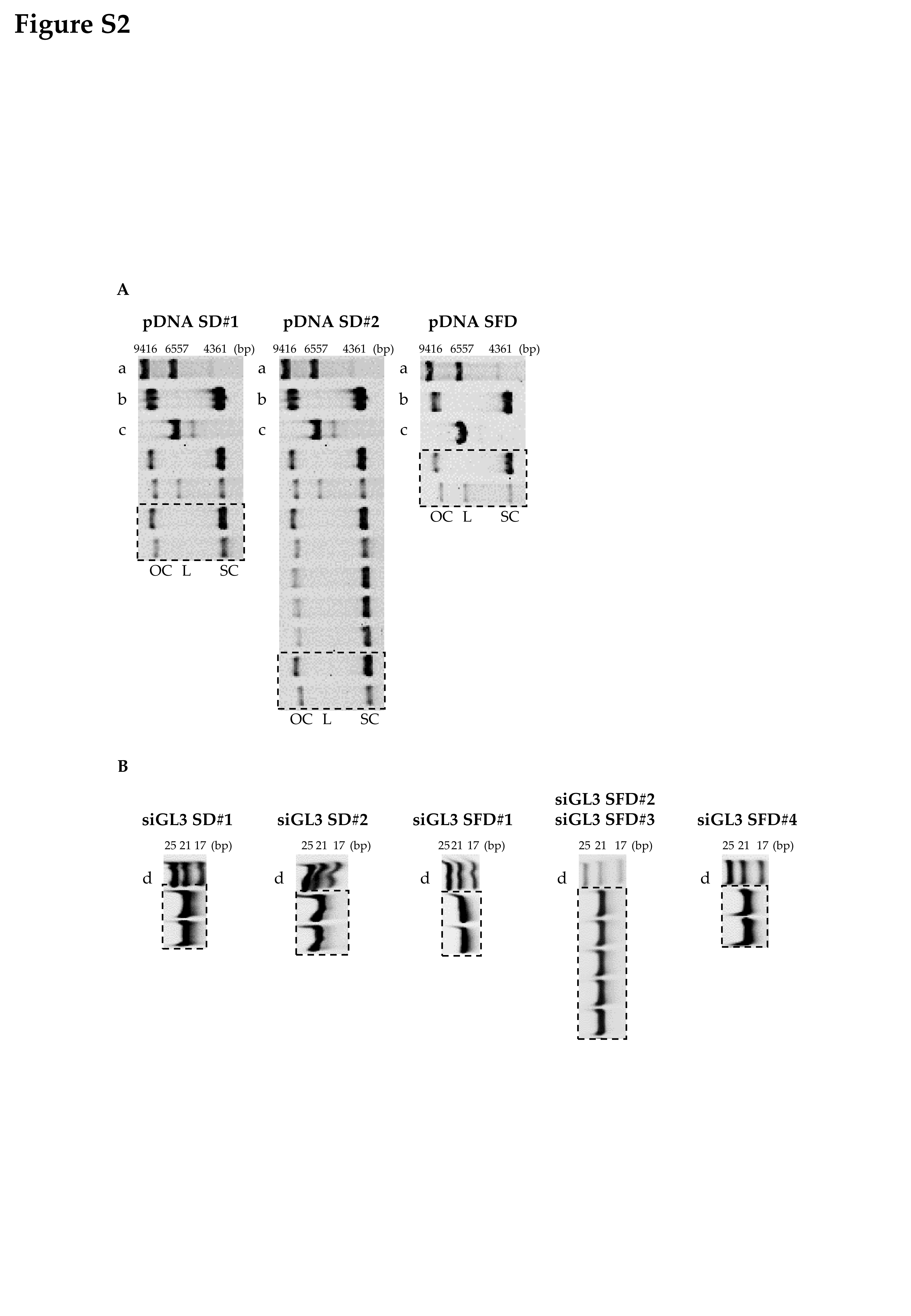

Supplement: Supplementary file 1 [file pharmaceutics-15-02786-s001.zip › Figure S2.tif]
